# The Effect of Artificial Liver Support System on Prognosis of HBV-Derived Hepatorenal Syndrome: A Retrospective Cohort Study

**DOI:** 10.1155/2022/3451544

**Published:** 2022-06-01

**Authors:** Xinyu Sheng, Jiaqi Zhou, Xiuyu Gu, Hong Wang

**Affiliations:** ^1^Department of Infectious Disease, Zhejiang Hospital, Hangzhou, China; ^2^Department of Respiration, The First Hospital of Jiaxing (The Affiliated Hospital of Jiaxing University), Jiaxing, Zhejiang, China; ^3^Department of Clinical Laboratory, The Affiliated Suzhou Hospital of Nanjing Medical University, Suzhou, Jiangsu, China

## Abstract

Hepatorenal syndrome (HRS) could occur when patients get decompensated liver cirrhosis. Meanwhile, hepatitis B virus (HBV) infection raises the risk of mortality of the end-stage liver diseases. As the artificial liver support system (ALSS) has been applied in liver failure, whether ALSS could benefit HBV-derived HRS remains uncertain. We retrospectively enlisted eligible HRS patients and compared the baseline characteristics and prognosis between HBV-derived HRS and non-HBV-derived HRS. Furthermore, propensity score matching (PSM) and Cox regression analyses were used to assess the beneficial effect of ALSS on HBV-derived HRS. In addition, a stratified analysis was carried out according to the degree of acute kidney injury (AKI) and the number of organ failures to observe in which populations ALSS can obtain the most excellent therapeutic effect. 669 patients were diagnosed as HRS, including 298 HBV negative and 371 HBV positive. Baseline characteristics were different between patients with HBV positive and HBV negative. HBV-derived HRS has higher 28-day mortality, though without a statistical difference. After PSM, 50 patients treated with ALSS and 150 patients treated with standard medical treatment (SMT) constituted a new cohort for the following analysis. We found that ALSS could significantly benefit HRS patients (*P* = 0.025). Moreover, the median survival time of patients treated with ALSS was longer than those treated with SMT. INR, neutrophil percentage, and treatment with ALSS were independent predictive factors for short-term mortality in HBV-derived HRS. The stratified analysis showed that ALSS could reduce the 28-day mortality of patients with HBV-derived HRS, especially those in AKI stage 3 and with organ failure ≥ 2. Additionally, serum bilirubin was significantly lower after ALSS, and the alteration of INR and creatinine were independent predictive elements for the mortality of HBV-derived HRS. HBV-derived HRS is more severe than non-HBV-derived HRS and has a worse prognosis. ALSS could reduce the short-term mortality of patients with HBV-derived HRS, especially those in AKI stage 3 and with organ failure ≥ 2. INR and the change of creatinine and INR could predict the prognosis of HBV-derived HRS. ChiCTR2200060123.

## 1. Introduction

Patients with cirrhosis are more prone to acute kidney injury (AKI). According to reports, 20% of the hospitalized patients with cirrhosis may get AKI [1]. Hepatorenal syndrome (HRS) means a progressive renal dysfunction in cirrhosis patients and high mortality in a brief time, which is one of the severe complications of decompensated cirrhosis [2, 3]. The probability of patients with liver cirrhosis and ascites developing to HRS within five years is up to 40% [4]. Given the unclear diagnosis, treatment strategies for HRS are inaccurate, which may result in high mortality of HRS. Hepatitis B virus (HBV) has been threatening health for many years. There are more than 350 million HBV carriers all around the world.

Millions of people suffer from HBV-related liver diseases every year [5]. Although the number of HBV-related liver diseases has been decreasing with the prevalence of HBV vaccines, it still brings significant challenges to many countries' medical and health services, including China. HBV infection has been proved to be associated with hepatitis, cirrhosis, and even hepatocellular carcinoma, which could cause an unwell prognosis [6–8]. A multicenter descriptive study has revealed that the original characteristics of COVID-19 cases combined with HBV infection were a higher rate of liver injury, coagulation disorders, severe/critical tendency, and increased susceptibility [9]. Considering the unique pathophysiology of HRS with extrahepatic manifestations, it is essential to pay more attention to HBV-related HRS. The most effective treatment for HRS is liver transplantation, but due to insufficient donors and economic constraints, the proportion of liver transplantation is small. Thus, finding a cost-effective treatment that can effectively improve the survival of HBV-derived HRS has become the top priority.

Various artificial liver support systems (ALSSs) have been widely used during past decades [8, 9]. ALSS could remove harmful substances from the patient's body and supplement the substances needed in the body through physical means, using the unique biofilm and the adsorption of chemical substances [10]. ALSSs have several types, and Professor Li's team launched a novel ALSS named Li's artificial liver system (Li-ALS) which includes plasma exchange, charcoal hemoperfusion, plasma bilirubin absorption, charcoal plasma perfusion, hemofiltration, and hemodialysis and has been applied in China since the 1980s [11]. Many kinds of research have proved that it could benefit patients with end-stage liver diseases, especially HBV-related acute-on-chronic liver failure. However, studies on the effect of ALSS on HRS, especially HBV-derived HRS, are not abundant and whether ALSS could benefit this part of patients has been uncertain.

We conducted a multicenter, retrospective, and long-term study to evaluate the association between ALSS and HBV-derived HRS. And we use propensity score matching (PSM) to balance confounding variables.

## 2. Materials and Methods

### 2.1. Study Population and Data Collection

In this cohort study, we screened patients from four general hospitals from January 2011 to March 2021, including the First affiliated Hospital of Zhejiang University, Shulan Hospital, People's Hospital of Zhejiang Province, and People's Hospital of Shengzhou City. The patients with decompensated cirrhosis and acute renal injury were enrolled at admission. Demographic data and vital signs were obtained from medical records. And follow-up was tracked by phone or address. Considering the rapid progress of HRS, we recorded 28-day mortality as our primary outcome and change of laboratory indexes as a secondary outcome. All assays for serum biochemical parameters were operated with the same testing equipment. The study was approved by the Ethics Committee of the First Affiliated Hospital, Zhejiang University (No. 2019-1449-1), and developed according to the ethical guidelines of the Declaration of Helsinki.

### 2.2. Inclusion and Exclusion Criteria

HRS was identified according to the standard from the International Club of Ascites (ICA) in 2015. An increase in sCr ≥ 26.5 mmol/L (≥0.3 mg/dL) within two days or 1.5 times the baseline was AKI. Detailed stage information was listed. AKI stage 1: increase in sCr ≥ 26.5 mmol/L (≥0.3 mg/dL) or an increase in sCr ≥ 1.5-fold to 2-fold from baseline. AKI stage 2: increase in sCr > two to threefold from baseline. AKI stage 3: increase of sCr > threefold from baseline or sCr ≥ 353.6 mmol/L (4.0 mg/dL) with an acute increase ≥ 26.5 mmol/L (≥0.3 mg/dL) or received renal replacement therapy. HBV positive was defined as HBV surface antigen positive ≥ six months, serum HBV-DNA ≥ 20000 IU/mL, or liver biopsy indicating chronic hepatitis.

Exclusion criteria are as follows: (1) absence of ascites, (2) any benign or malignant carcinoma (37), (3) chronic renal injury, (4) liver transplantation or severe immunosuppression, (5) age < 18 years, (6) hospital stay was less one week, and (7) incomplete information. Also, patients lost to follow-up were excluded.

For ACLF grade 1, patients include those with single organ failure, mainly coagulation, circulatory, respiratory systems or kidney failure. For ACLF grade 2, patients include those with two organ failures. ACLF grade 3: in patients include those with 3 or more organ system failures.

Model 1 was adjusted for age and sex. Model 2 was adjusted for age, sex, neutrophils, alanine aminotransferase (ALT), albumin, serum bilirubin, COSSH-ACLFs, and international normalized ratio (INR).

Liver failure was defined as serum bilirubin ≥ 12 mg/dL, coagulation failure as INR ≥ 2.5, brain failure as hepatic encephalopathy grade ≥ 3 (West Haven criteria), and circulatory failure as the need for vasopressor therapy to maintain blood pressure [12].

### 2.3. Treatment

According to ICA-AKI diagnostic criteria, standard medical treatment (SMT) was applied according to the stage of AKI, including treatment of infections, plasma volume expansion, withdrawal of nephrotoxic or nonsteroidal anti-inflammatory drugs, and basic life support. ALSS treatment adopted Li-ALS. Li-ALS includes plasma exchange (PE), hemodialysis (HD), hemofiltration (HF), and hemoperfusion (HP). PE uses hollow fiber membrane separation technology to filter the toxin-containing plasma components (mainly protein-binding toxins) in the blood out of the membrane and discard them and place equal amounts of fresh frozen plasma and albumin with the blood in the membrane and return to the body together. HF uses a membrane with a larger pore size and relies on the pressure difference between the liquids on both sides of the membrane as the transmembrane pressure, mimicking the principle of glomerular filtration function and removing excess water and toxic substances from the blood by convection. HP uses a cylindrical perfusion device containing special activated carbon or resin particles to remove toxins or drugs in the blood by adsorption, and the perfused blood returns to the body through a catheter. HD mainly relies on the concentration gradient dispersion on both sides of the membrane to precipitate small water-soluble substances such as blood Cr and urea nitrogen, to correct water and electrolyte disorders and acid-base balance disorders. Patients receive Li-ALS treatment approximately 1-2 times a week until TB ≤ 5 mg/dL or persistent hyperbilirubinemia and coagulopathy improve, or until liver transplantation. When active bleeding or circulatory failure occurs, it needs to be stopped.

### 2.4. Statistical Analysis

Clinicopathological features were summarized using medians with interquartile ranges (IQRs) or frequencies with percentages, and biochemical parameters were compared using the Wilcoxon rank-sum, chi-squared, and Fisher exact test. The propensity score (PS) for ALSS was estimated using a logistic regression model with ALSS as the outcome. All 371 HBV-positive patients were included in the PS analytical cohort. The associations between ALSS and overall survival were evaluated using Cox regression models and summarized as hazard ratios (HRs) with 95% confidence intervals (CIs). The PS techniques employed propensity score matching (PSM). A propensity score matching (PSM) method was applied to compare the mortality between the patients treated with ALSS and SMT. Patients treated with ALSS were matched in a 1 : 3 ratio to patients treated with SMT only using a method based on the logit of the PS. Statistical analyses were performed with the aid of R ver. 4.0.5 (R Foundation for Statistical Computing, Vienna, Austria). All tests were two-sided, and a *P* value < 0.05 was considered statistically significant.

## 3. Results

### 3.1. HBV-Derived HRS Is More Severe than Non-HBV-Derived HRS

**Figure 1:**
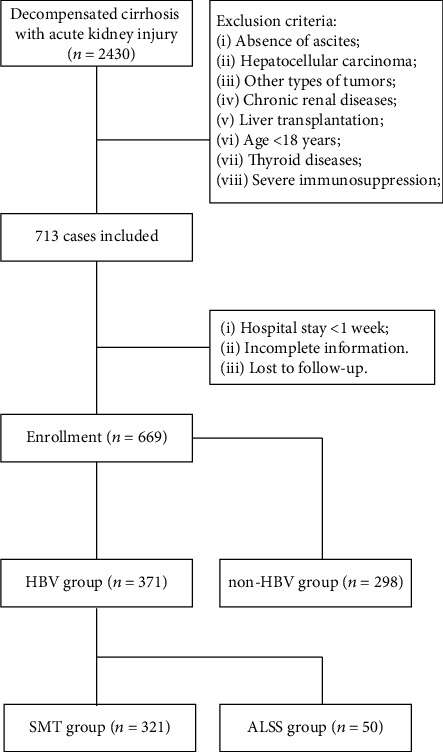
Screening and enrollment of patients. ALSS: artificial liver support system; SMZ: standard medical treatment.

**Figure 2:**
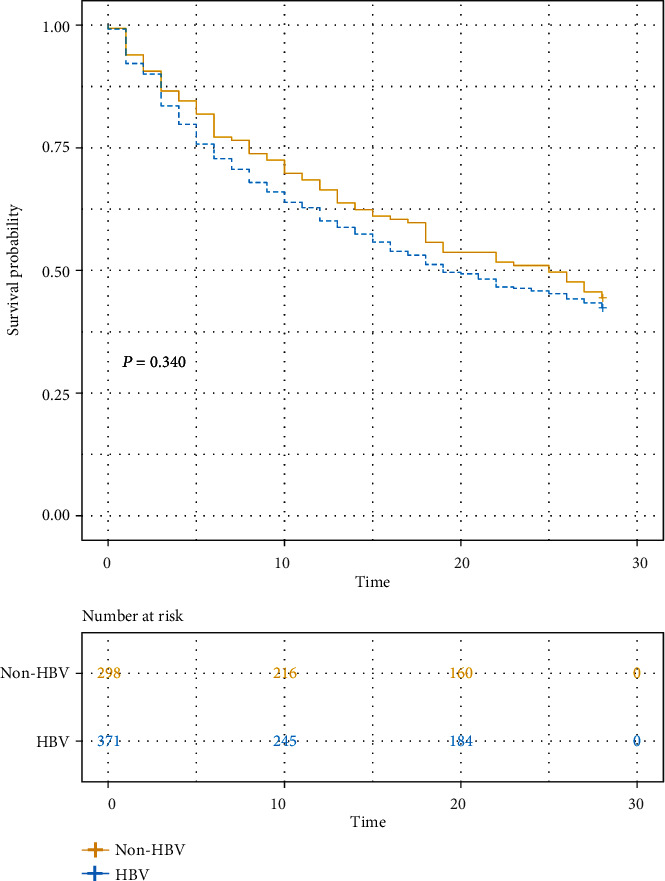
Kaplan–Meier curves of HBV-derived HRS and non-HBV-derived HRS. HRS: hepatorenal syndrome; HBV: hepatitis B virus.

A total of 669 patients were diagnosed as HRS used by inclusion and exclusion criteria. 669 HRS patients, including 298 HBV negative and 371 HBV positive, were enrolled for the subsequent analysis (Figure 1). The baseline characteristics of both cohorts are listed in Table 1. No significant differences in the heart rate, neutrophil percentage, globulin, cystatin C, urea, potassium, kidney failure, and other indexes were found between the HBV-derived HRS and non-HBV-derived HRS groups. However, the age of onset of the HBV-derived HRS cohort was lower, and the proportion of male patients was higher. Also, the coagulation and liver function of the HBV-derived HRS cohort were worse, along with elevated serum bilirubin, ALT, and AST levels. Thus, it could be inferred that the pathology is different between the HBV-derived HRS cohort and non-HBV-derived cohort. In this way, patients with HBV-derived HRS should be paid more attention. Moreover, KM curves showed that HBV-derived HRS has higher 28-day mortality, though with no statistical differences (*P* = 0.340) (Figure 2). Considering worse liver function and prognosis, HBV-derived HRS deserves further research.

### 3.2. Baseline Characteristics of Patients with HBV-Derived HRS after PSM

321 patients received SMT, and 50 patients received ALSS treatment in the whole HBV-derived HRS cohort. Generally, there were significant differences in ascitic grade, urea, creatinine, serum bilirubin, CTP score, etc., between HRS patients who received SMT and ALSS treatment (Table 2). Considering the selection bias of the retrospective study, we adopted the PSM method to balance the confounding factors. Patients with SMT and ALSS treatment were matched in a ratio of 3 to 1 and then integrated into a new cohort. Several indexes were balanced between two cohorts while the other indexes still differed, indicating that the baseline characteristics are quite different (Table 3).

### 3.3. ALSS Reduce the Mortality of HBV-Derived HRS

**Figure 3:**
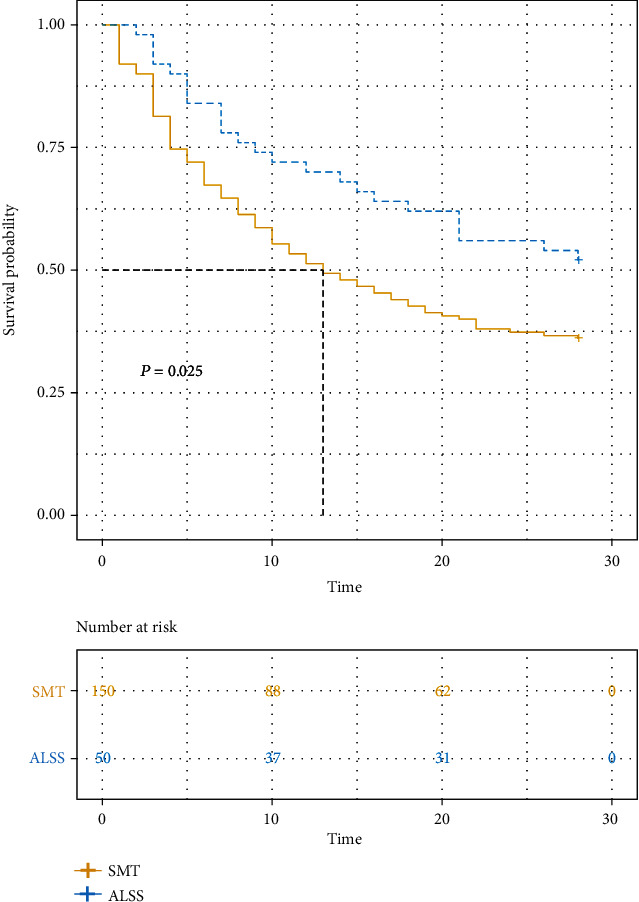
Kaplan–Meier curves of HBV-derived HRS treated with ALSS and SMT only after PAM. ALSS: artificial liver support system; HBV: hepatitis B virus; HRS: hepatorenal syndrome; SMZ: standard medical treatment.

To further evaluate the effect of ALSS on the prognosis of HBV-derived HRS, we conducted KM curves in a new cohort after PSM. Finally, we found ALSS could significantly benefit HRS patients (*P* = 0.025). The median survival time of patients in SMT group was 13 days, while those treated with ALSS were more than 28 days (Figure 3).

Additionally, in univariate analysis, HE degree III, ascitic, ALT, AST, cystatin C, urea, creatinine, iMELD, MELDs, CLIF-ACLFs, CLIF-SOFAs, COSSH-ACLFs, organ failures, and treated with ALSS were associated with 28-day mortality. When combined with multivariate analysis, eventually, INR, neutrophil percentage, and treated with ALSS were independent predictive factors for 28-day mortality in HBV-derived HRS. The mortality of patients treated with ALSS was 0.6 times that of those without ALSS, which could considerably prolong the life of patients (Table 4).

### 3.4. ALSS Could Acquire More Survival Benefit in AKI Stage 3

**Figure 4:**
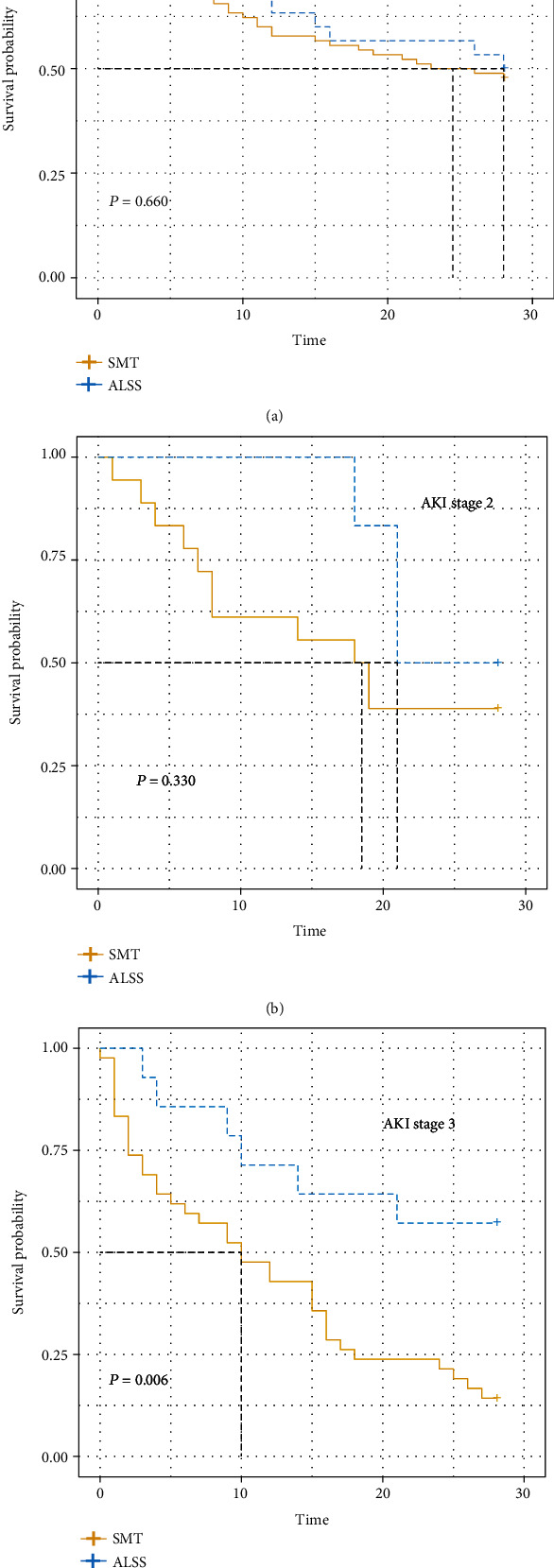
Kaplan–Meier curves of mortality of ALSS in different AKI degrees. (a) The effect of ALSS in AKI stage 1. (b) The effect of ALSS in AKI stage 2. (c) The effect of ALSS in AKI stage 3. ALSS: artificial liver support system; AKI: acute kidney injury.

According to the definitions of diagnosis of HRS from the International Club of Ascites (ICA-AKI), the severity of AKI could be classified into three stages. In this way, patients could be divided into three stages, namely, AKI stage 1, AKI stage 2, and AKI stage 3. The baseline characteristics of AKI stage 1, AKI stage 2, and AKI stage 3 are shown in Table S1. Similarly, some indexes were different between patients with ALSS and those without ALSS. The Cox regression model was developed to figure out the effect of ALSS on prognosis in different AKI stages. Here, we developed three kinds of models, crude model, model 1, and model 2. Finally, in all three models, ALSS could acquire survival benefit in AKI stage 3, and the mortality of patients treated with ALSS was 0.37, 0.34, and 0.29 times that of those without ALSS in the crude model, model 1, and model 2, respectively (Table 5). In other words, ALSS could reduce the population's mortality rate by 2/3 in the AKI stage 3 cohort. Nevertheless, in other patients, the 28-day mortality remained similar between patients with/without ALSS, especially in the AKI stage 1 cohort. Both the results of KM curves and Cox regression analysis support this conclusion (Figure 4). The mortality of patients with ALSS was much lower than that of patients without ALSS in the AKI stage 3 cohort (*P* = 0.006). The median survival time was 10 days in patients without ALSS, while the median survival time was more than 28 days in patients with ALSS. In total, ALSS could greatly benefit patients in severe HBV-derived HRS.

### 3.5. ALSS Could Acquire More Survival Benefit with Organ Failure ≥ 2

**Figure 5:**
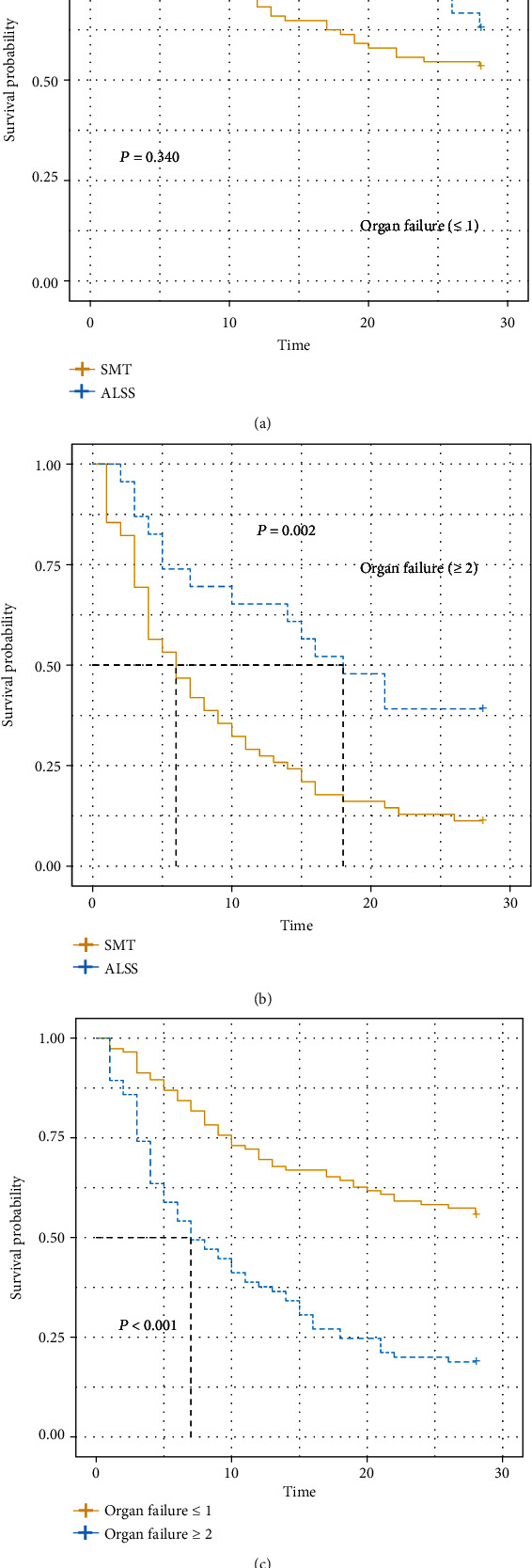
Kaplan–Meier curves of mortality of ALSS in patients with organ failures ≥ 2 and ≤1. (a) The effect of ALSS in patients with organ failure ≤ 1. (b) The effect of ALSS in patients with organ failure ≥ 2. (c) The effect of the number of organ failures on mortality. ALSS: artificial liver support system.

According to the number of organ failures, patient with HBV-derived HRS could be divided into two groups; the number of organ failures ≤ 1 and ≥2. The baseline characteristics are listed in Table S2. In patients with 0 or 1 organ failure, ALT, AST, creatinine, and urea were different between patients with and without ALSS. In patients with more organ failures, those two groups differed in heart rate, cystatin C, iMELD, MELDs, and COSSH-ACLFs. Given the variety, the effect of ALSS on prognosis was evaluated in Table 5. Finally, ALSS could reduce the mortality in patients with more than two organ failures by almost half in all three models. From Figure 5, we could find that patients with more organ failures are at high risk of mortality (*P* = 0.002) but could benefit from ALSS. Combined with the results in different AKI stages, ALSS could significantly reduce the mortality of severe HBV-derived HRS patients.

### 3.6. Patients Treatment with ALSS Have Lower Scores and Mortality

**Figure 6:**
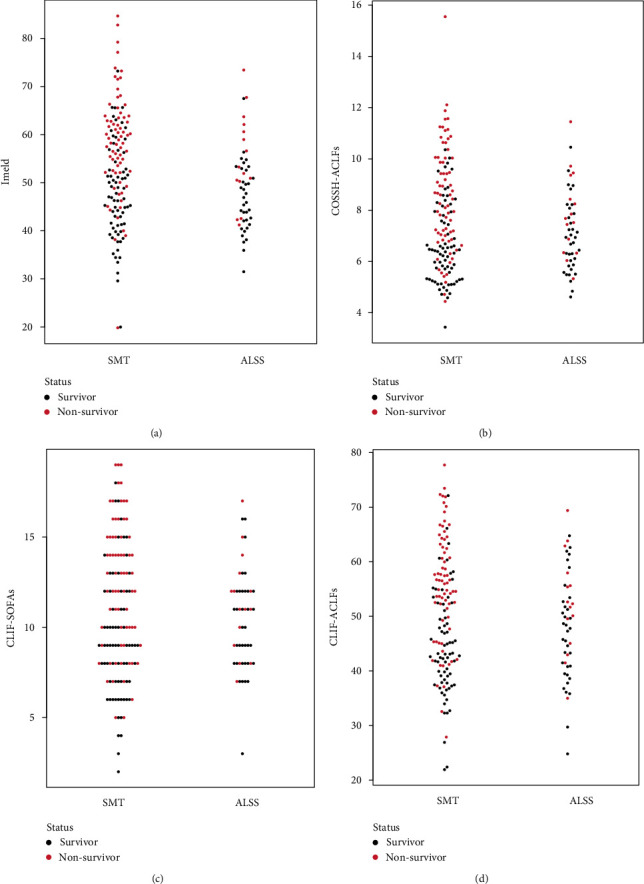
Score distribution in patients treated with ALSS or SMT only. ALSS: artificial liver support system; SMZ: standard medical treatment.

Figure 6 displayed various score systems, including iMELD, CLIF-ACLFs, CLIF-SOFAs, and COSSH-ACLFs, after patients were treated with ALSS or SMT only. The iMELDs was much higher in patients treated with SMT rather than ALSS, while nonsurvivors were concentrated in the higher iMELD part. Consistently, this trend remained the same when patients were evaluated by CLIF-ACLFs, CLIF-SOFAs, and COSSH-ACLFs. As all the four scores were found to be associated with mortality of HBV-derived HRS, generally, it can be inferred that ALSS might help reduce the scores and benefit the prognosis of HBV-derived HRS.

### 3.7. The Change of INR and Creatinine Were Independent Predictive Factors for the Mortality of HBV-Derived HRS

The ALT, serum bilirubin, creatinine, INR, and neutrophil were monitored both pre-ALSS and post-ALSS treatment. Serum bilirubin was significantly decreased after ALSS treatment (*P* = 0.004), while ALT, creatinine, INR, and neutrophil percentage remained at the same level (Table 6). To further assess the effect of the change of indexes on 28-day mortality, we included the change of serum bilirubin, ALT, neutrophil percentage, INR, and creatinine into Cox regression analysis. We found that the change of INR and creatinine were independent predictive factors for the prognosis of HBV-derived HRS (*P* = 0.020 and 0.016, respectively) (Table 7).

## 4. Discussion

This study retrospectively enrolled HRS patients from multiple centers in the past ten years and obtained 28-day mortality through telephone follow-up. From the total patients, we found the distinct characteristics between HBV-derived HRS and non-HBV-derived HRS and worse prognosis in those with HBV positive. Then, we balanced the selection bias through PSM and concluded that ALSS could improve the prognosis of HBV-derived HRS whenever in various Cox regression models. As for hierarchical analysis, ALSS could greatly benefit patients in AKI stage 3 and with ≥ two organ failures. Finally, serum bilirubin was reduced after ALSS treatment, and the change of INR and creatinine could predict the 28-day mortality of HBV-derived HRS. Eventually, ALSS could improve the prognosis of HBV-derived HRS, especially severe HRS.

The HBV infection rate has been high in China [13, 14]. Although newborns are generally vaccinated against HBV, the current situation of HBV infection is still severe. Specifically, HBV infection still accounts for a large proportion of the causes of HRS; 371 out of 669 patients were HBV positive in this study. Patients with HBV positive had higher INR, ALT, AST, serum bilirubin, and proportion of coagulation failure than those with HBV negative, which is not conducive to the prognosis of the HRS. Consistent with the previous study, patients with HBV positive are at risk of higher mortality in the KM curve. The previous view believed that HRS is only a kind of renal dysfunction and the structure of the kidney is normal. However, electron microscopy studies on kidneys obtained from HRS patients shortly after death have demonstrated renal tubular tears and the presence of dark bodies in mitochondria [15]. Besides, a particular lesion involving reflux of the proximal convoluted tubule epithelium into the Bowman space has also been described in autopsy specimens from patients with HRS [16]. Like hepatitis C virus (HCV) infection, the pathogenetic role of HBV infection has been documented primarily by the demonstration of hepatitis B antigen-antibody complexes in the renal lesions via immunofluorescence microscopy [4, 17]. In this way, HBV-derived HRS is recommended for more attention.

Several indexes were different, including ascitic, hemoglobin, cystatin C, urea, creatinine, serum bilirubin, and CTP score between patients treated with ALSS and SMT only. After being balanced by PSM, some of them remained at the same level between the two groups, indicating the PSM method's efficacy. To figure out the association between ALSS and prognosis of HBV-derived HRS, we did survival analysis, and it showed that the median survival time of patients with ALSS is longer than those treated only with SMT, and ALSS could reduce mortality.

We enrolled various indexes into univariate Cox regression analysis to further reveal ALSS and predictive factors for 28-day mortality. Then, we found different degrees of ascitic, heart rate, INR, neutrophil percentage, ALT, AST, cystatin C, urea, and creatinine were associated with the prognosis of HBV-derived HRS [18]. Liver function, including the degree of ascitic, ALT, AST, and cystatin C, and renal function, including urea and creatinine, account for the most factors related to prognosis [19]. Additionally, standard score systems, MELDs, iMELD, CTP, CLIF-ACLFs, CLIF-SOFA, and COSSH-ACLFs, were calculated according to mainly liver function. Thus, it is reasonable that these score systems are related to the prognosis [20–23]. When selected for multivariate Cox regression, INR and neutrophil percentage are independent predictive factors for 28-day mortality. One of the elements to assess the severity of advanced liver diseases is INR for decades [24]. Usually, higher INR means blood coagulation dysfunction and may result in an unwell prognosis of advanced liver diseases, including HRS. Neutrophil percentage is positively correlated with the severity of systemic inflammation. Advanced liver disease is often accompanied by bacterial infections, increasing the percentage of neutrophils [25, 26]. We found that it can predict the mortality of HRS. As it is convenient and readily available, neutrophil percentage could serve as a monitor factor. Moreover, ALSS could significantly reduce mortality. In this way, we could treat patients with ALSS and use INR and neutrophil percentage as monitor factors to give more survival benefits to patients with HBV-derived HRS.

According to the definition of AKI from the International Club of Ascites (ICA-AKI), there are three stages of AKI [12]. As the degree of AKI could influence the outcome, we wonder whether ALSS could benefit all degrees of AKI. We developed three models adjusted by various variables. Finally, ALSS greatly benefits patients in AKI stage 3. This may result from the working principle of ALSS, which can take away metabolic waste and replace it with normal plasma. This can quickly correct the fluid balance and restore liver and kidney functions. Patients in AKI stage 1 and AKI stage 2 may regulate their internal environment disorders through their adjustment ability.

ACLF is also a common advanced liver disease with rapid liver dysfunction and high mortality [27]. There are many similarities between HRS and ACLF; for example, ACLF patients often have kidney damage, continued collection of various metabolites and toxins and systemic inflammation, which means that treatment for ACLF could also help patients with HRS. Non-HBV-ACLF patients were confirmed to have a good prognosis [14, 28, 29]. The effect of ALSS on HRS has been uncertain before; however, ALSS could significantly reduce mortality of ACLF [30–33]. A study of 132 patients with HBV-ACLF revealed that ALSS could better improve the short-term survival of HBV-ACLF patients than SMT alone, especially in those with HBV-ACLF with infection [34, 35]. This is consistent with our results that ALSS could significantly promote survival of patients with HBV-derived HRS, especially those in AKI stage 3.

ACLF degree is defined mainly according to the degree of organ failures. It is artificially separated into ACLF-1, ACLF-2, and ACLF-3 according to the number of organ failures, and this classification is significantly associated with the prognosis of ACLF. Similarly, we divided our patients into 2 groups in the same way. Finally, ALSS could give great benefit to those with ≥2 organ failures. The mortality of patients treated with SMT only is about 3 times that of patients treated with ALSS. As described before, organ failures were associated with the severity and outcome of ACLF [32, 36]. Also, according to the mechanism of ALSS, it could rapidly improve organ function. The consistency of the above two stratified analyses illustrates the reliability of the results. We can conclude that ALSS can reduce the mortality of HRS patients, especially those with multiple organ failures and severe renal dysfunction.

MELDs has been developed to evaluate the liver function of liver diseases. It contains total serum bilirubin, INR, and creatinine. INR and the change of creatinine and INR could predict the prognosis of HBV-derived HRS. Patients with higher INR and creatinine may get a worse outcome. According to the bee swarm plot related to iMELD, CLIF-SOFAs, CLIF-ACLF, and COSSH-ACLF, the scores of all four systems are higher in patients treated with SMT only. Although scoring systems above could predict the mortality of HRS, the severe complication of decompensated cirrhosis, a novel predictive tool that specifically predicts the mortality of HRS is needed. Our team has launched a novel tool named GIMNS, which combines neutrophil percentage and INR, to predict mortality of HRS [37].

Indexes including ALT, serum bilirubin, creatinine, INR, and neutrophil percentage were reassessed after ALSS treatment. The level of serum bilirubin decreased while the others remained the same. The small sample size of patients could cause this as some information was missing due to retrospective data. But the change of INR and creatinine are proved to be predictive factors for 28-day mortality in patients treated with ALSS. Decreased creatinine and INR after ALSS treatment may represent a better prognosis of HBV-derived HRS. Our study also has some limitations. First, this is a retrospective cohort study, and some selection biases exist. In this way, we adopted PSM analysis to balance the confounding variables and enrolled four general hospitals to increase the sample size. Second, we diagnosed HRS according to the latest criteria from ICA-AKI to improve the accuracy of diagnosis. But definitions for the diagnosis of HRS have not been clear. The patients we enrolled may contain those with acute tubular necrosis (ATN). Thus, more clinical trials on HRS should be carried out to define HRS better. Finally, our study cohort did not adopt urine output as a diagnostic indicator. We would add this index in the following prospective cohort study.

## 5. Conclusions

In summary, HBV-derived HRS is more severe than non-HBV-derived HRS and has a worse prognosis. ALSS could reduce the 28-day mortality of patients with HBV-derived HRS, especially those in AKI stage 3 and with organ failure ≥ 2. INR and the change of creatinine and INR could predict the prognosis of HBV-derived HRS.

## Figures and Tables

**Table 1:** Characteristics between HBV-derived HRS and non-HBV-derived HRS.

Variates	Non-HBV-derived HRS	HBV-derived HRS	*P* value
*n*	298	371	
Age (year)	62.54 ± 11.66	57.82 ± 12.38	<0.001
Male sex	208 (69.80)	282 (76.00)	0.086
Degree of HE			0.998
Without HE (%)	164 (55.0)	202 (54.4)	
I	42 (14.1)	55 (14.8)	
II	26 (8.7)	34 (9.2)	
III	22 (7.4)	26 (7.0)	
IV	44 (14.8)	54 (14.6)	
Ascitic (%)			0.222
Grade 1	54 (18.10)	70 (18.90)	
Grade 2	84 (28.2)	110 (29.6)	
Grade 3	120 (40.3)	122 (32.9)	
Missing data	40 (13.4)	69 (18.6)	
MAP (mmHg)	93.01 ± 16.06	95.97 ± 16.76	0.021
HR	85.43 ± 15.57	87.14 ± 16.10	0.166
INR	1.75 (1.40-1.99)	1.84 (1.51-2.42)	<0.001
Neutrophil (%)	74.27 ± 11.83	74.80 ± 11.93	0.566
Albumin (g/L)	28.50 ± 5.59	29.25 ± 5.46	0.079
Globulin (g/L)	29.44 ± 9.09	28.81 ± 8.98	0.368
ALT (U/L)	36.50 (21.00-106.00)	50.50 (24.00-125.00)	0.053
AST (U/L)	63.50 (34.50-98.75)	80.50 (41.00-164.50)	0.006
Hemoglobin (g/L)	93.41 ± 26.13	101.05 ± 26.88	<0.001
Cystatin C (mg/L)	2.16 (1.56-3.53)	1.95 (1.34-3.24)	0.197
Urea (mmol/L)	14.53 (9.05-24.40)	13.30 (7.77-23.10)	0.284
Creatinine (mg/dL)	1.83 (1.03-2.71)	1.62 (0.93-2.64)	0.656
Serum bilirubin (mg/dL)	11.75 (2.28-22.28)	13.68 (3.17-24.88)	0.073
GGT (U/L)	70.00 (32.00-152.00)	59.00 (34.00-122.00)	0.107
Potassium (mmol/L)	4.20 ± 0.81	4.63 ± 0.72	0.281
Sodium (mmol/L)	134.45 ± 6.12	133.95 ± 6.70	0.049
MELDs	26.21 ± 8.09	27.55 ± 9.21	0.742
iMELD	50.86 ± 10.24	51.14 ± 11.21	0.179
CTP	10.95 ± 1.92	11.18 ± 1.92	0.122
CLIF-ACLFs	50.57 ± 10.14	49.69 ± 10.57	0.278
CLIF-SOFAs	10.01 ± 3.49	10.14 ± 3.56	0.620
COSSH-ACLFs	7.13 ± 1.63	7.19 ± 1.85	0.648
Liver failure	146 (49.0)	198 (53.4)	0.295
Coagulation failure	42 (14.1)	84 (22.6)	0.007
Cerebral failure	66 (22.1)	80 (21.6)	0.930

ALT: alanine aminotransferase; CLIF-SOFA: Chronic Liver Failure-Sequential Organ Failure Assessment; COSSH-ACLF: Chinese Group on the Study of Severe Hepatitis B-Acute-on-Chronic Liver Failure; HBV: hepatitis B virus; INR: international normalized ratio; MAP: mean arterial pressure; MELD: Model for End-Stage Liver Disease.

**Table 2:** Characteristics between SMT and ALSS before PSM.

Variates	SMT	ALSS	*P* value
*n*	321	50	
Age (year)	58.46 ± 12.17	53.70 ± 13.05	0.011
HBV-DNA (log copies/mL)	5.1 ± 1.5	5.1 ± 1.6	0.986
Male sex	240 (74.80)	42 (84.00)	0.213
Degree of HE			0.322
Without HE	179 (55.80)	23 (46.00)	
I	49 (15.30)	6 (12.00)	
II	27 (8.40)	7 (14.00)	
III	20 (6.20)	6 (12.00)	
IV	46 (14.30)	8 (16.00)	
Ascitic (%)			0.003
Grade 1	58 (17.00)	12 (24.00)	
Grade 2	87 (27.10)	23 (46.00)	
Grade 3	115 (35.80)	7 (14.00)	
Missing data	61 (19.00)	8 (16.00)	
MAP (mmHg)	95.83 ± 17.02	96.88 ± 15.14	0.682
HR	87.40 ± 16.47	85.46 ± 13.45	0.428
INR	1.84 (1.46-2.33)	2.08 (1.74-2.63)	0.137
WBC (10^9^/L)	7.40 (4.98-11.40)	6.55 (5.05-9.58)	0.072
Neutrophil (%)	75.29 ± 12.04	71.68 ± 10.79	0.047
Albumin (g/L)	29.21 ± 5.60	29.55 ± 4.54	0.686
Globulin (g/L)	28.73 ± 8.99	29.30 ± 8.97	0.677
ALT (U/L)	44.50 (22.00-104.20)	114.00 (54.75-253.50)	0.060
AST (U/L)	70.00 (39.00-154.20)	144.50 (92.50-282.00)	0.202
Hemoglobin (g/L)	98.80 ± 26.44	115.22 ± 25.51	<0.001
Cystatin C (mg/L)	2.14 (1.53-3.41)	1.24 (0.92-1.70)	0.002
Urea (mmol/L)	14.40 (8.50-23.75)	7.95 (4.23-14.50)	0.006
Creatinine (mg/dL)	1.75 (1.01-2.75)	0.93 (0.71-1.61)	0.001
Serum bilirubin (mg/dL)	12.61 (2.51-24.09)	20.07 (10.64-28.13)	0.011
GGT (U/L)	59.00 (33.00-123.00)	61.50 (45.75-99.50)	0.393
Potassium (mmol/L)	4.30 ± 0.91	4.19 ± 0.74	0.627
Sodium (mmol/L)	133.77 ± 6.91	135.06 ± 5.10	0.208
MELDs	27.55 ± 9.54	27.59 ± 6.72	0.979
iMELD	51.44 ± 11.53	49.15 ± 8.74	0.179
CTP	11.08 ± 1.96	11.82 ± 1.52	0.011
CLIF-ACLFs	49.87 ± 10.71	48.54 ± 9.65	0.408
CLIF-SOFAs	10.09 ± 3.67	10.46 ± 2.79	0.499
COSSH-ACLFs	7.19 ± 1.89	7.20 ± 1.51	0.988
Liver failure	163 (50.80)	35 (70.00)	0.017
Coagulation failure	68 (21.20)	16 (32.00)	0.129
Cerebral failure	66 (20.60)	0 (0.00)	0.315
28-day mortality	190 (59.20)	24 (48.00)	0.182

ALT: alanine aminotransferase; CLIF-SOFA: Chronic Liver Failure-Sequential Organ Failure Assessment; COSSH-ACLF: Chinese Group on the Study of Severe Hepatitis B-Acute-on-Chronic Liver Failure; HBV: hepatitis B virus; INR: International normalized ratio; MAP: Mean arterial pressure; MELD: Model for End-Stage Liver Disease.

**Table 3:** Characteristics between SMT and ALSS after PSM.

Variates	SMT	ALSS	*P* value
n	150	50	
Age (year)	54.21 ± 11.59	53.70 ± 13.05	0.796
HBV-DNA (log copies/mL)	5.1 ± 1.7	5.1 ± 1.6	0.990
Male sex	124 (82.70)	42 (84.00)	1.000
Degree of HE			0.109
Without HE	81 (54.00)	23 (46.00)	
I	24 (16.00)	6 (12.00)	
II	7 (4.70)	7 (14.00)	
III	9 (6.00)	6 (12.00)	
IV	29 (19.30)	8 (16.00)	
Ascitic (%)			0.007
Grade 1	30 (20.00)	12 (24.00)	
Grade 2	37 (24.70)	23 (46.00)	
Grade 3	52 (34.70)	7 (14.00)	
Missing data	31 (20.70)	8 (16.00)	
MAP (mmHg)	95.82 ± 16.05	96.88 ± 15.14	0.683
HR	87.65 ± 16.61	85.46 ± 13.45	0.399
INR	2.01 (1.68-2.82)	2.08 (1.74-2.63)	0.536
WBC (10^9^/L)	7.15 (4.70-10.38)	6.55 (5.05-9.58)	0.241
Neutrophil (%)	72.88 ± 13.18	71.68 ± 10.79	0.563
Albumin (g/L)	29.30 ± 5.68	29.55 ± 4.54	0.777
Globulin (g/L)	28.66 ± 8.66	29.30 ± 8.97	0.654
ALT (U/L)	57.00 (25.50-127.00)	114.00 (54.75-253.50)	0.273
AST (U/L)	90.50 (49.75-205.00)	144.50 (92.50-282.00)	0.511
Hemoglobin (g/L)	99.82 ± 26.30	115.22 ± 25.51	<0.001
Cystatin C (mg/L)	2.14 (1.44-3.44)	1.24 (0.92-1.70)	0.007
Urea (mmol/L)	14.81 (6.75-23.82)	7.95 (4.26-14.50)	0.001
Creatinine (mg/dL)	1.80 (1.01-2.95)	0.93 (0.71-1.61)	<0.001
Serum bilirubin (mg/dL)	14.12 (3.95-25.28)	20.07 (10.64-28.13)	0.079
GGT (U/L)	50.00 (33.00-119.00)	61.50 (45.75-99.50)	0.501
Potassium (mmol/L)	4.39 ± 0.95	4.19 ± 0.74	0.480
Sodium (mmol/L)	132.97 ± 7.69	135.06 ± 5.10	0.074
MELDs	29.91 ± 9.54	27.59 ± 6.72	0.113
iMELD	53.09 ± 12.04	49.15 ± 8.74	0.034
CTP	11.47 ± 1.98	11.82 ± 1.52	0.259
CLIF-ACLFs	49.90 ± 11.41	48.54 ± 9.65	0.450
CLIF-SOFAs	10.83 ± 3.84	10.46 ± 2.79	0.527
COSSH-ACLFs	7.56 ± 2.10	7.20 ± 1.51	0.260
Liver failure	84 (56.00)	35 (70.00)	0.114
Coagulation failure	50 (33.30)	16 (32.00)	1.000
Cerebral failure	38 (25.30)	14 (28.00)	0.852
28-day mortality	96 (64.00)	24 (48.00)	0.067

ALT: alanine aminotransferase; CLIF-SOFA: Chronic Liver Failure-Sequential Organ Failure Assessment; COSSH-ACLF: Chinese Group on the Study of Severe Hepatitis B-Acute-on-Chronic Liver Failure; HBV: hepatitis B virus; INR: international normalized ratio; MAP: mean arterial pressure; MELD: Model for End-Stage Liver Disease.

**Table 4:** Univariate and multivariate Cox regression.

Variates	Univariate cox regression		Multivariate cox regression	
	HR (95% CI)	*P* value	HR (95% CI)	*P* value
Age (year)	0.99 (0.99-1.02)	0.329	1.02 (1.00-1.03)	0.069
Male sex	1.01 (0.62-1.63)	0.981	1.24 (0.76-2.03)	0.396
Degree of HE				
Without HE	Ref.			
I	1.57 (0.92-2.66)	0.096		
II	1.42 (0.70-2.90)	0.329		
III	2.40 (1.28-4.52)	0.007		
IV	2.67 (1.69-4.21)	<0.001		
Ascitic (%)				
Grade 1	Ref.			
Grade 2	0.41 (0.19-0.86)	0.019		
Grade 3	0.48 (0.23-1.00)	0.051		
Missing data	0.46 (0.21-0.99)	0.049		
MAP (mmHg)	0.99 (0.98-1.00)	0.180		
HR	1.02 (1.01-1.03)	<0.001		
INR	1.82 (1.56-2.02)	<0.001	1.61 (1.37-1.89)	<0.001
WBC (10^9^/L)	1.09 (1.06-1.12)	<0.001	1.03 (0.99-1.07)	0.104
Neutrophil (%)	1.05 (1.04-1.07)	<0.001	1.03 (1.01-1.05)	0.003
Albumin (g/L)	0.98 (0.94-1.01)	0.168	0.98 (0.94-1.02)	0.257
Globulin (g/L)	1.01 (0.99-1.03)	0.463		
ALT (U/L)	1.00 (1.00-1.00)	0.003		
AST (U/L)	1.00 (1.00-1.00)	0.001		
Hemoglobin (g/L)	1.00 (1.00-1.01)	0.219		
Cystatin C (mg/L)	1.25 (1.05-1.49)	0.012		
Urea (mmol/L)	1.01 (1.00-1.02)	0.012		
Creatinine (mg/dL)	1.00 (1.00-1.00)	0.046		
Serum bilirubin (mg/dL)	1.00 (1.00-1.00)	<0.001	1.00 (1.00-1.00)	0.104
GGT (U/L)	1.00 (1.00-1.00)	0.952		
Potassium (mmol/L)	0.99 (0.96-1.02)	0.535		
Sodium (mmol/L)	0.96 (0.94-0.98)	<0.001		
MELDs	1.10 (1.07-1.12)	<0.001		
iMELD	1.08 (1.06-1.10)	<0.001		
CTP	1.48 (1.32-1.67)	<0.001		
CLIF-ACLFs	1.08 (1.06-1.10)	<0.001		
CLIF-SOFAs	1.22 (1.16-1.28)	<0.001		
COSSH-ACLFs	1.47 (1.35-1.60)	<0.001		
Organ failure				
Liver failure	2.07 (1.40-3.07)	<0.001		
Coagulation failure	2.87 (1.99-4.13)	<0.001		
Cerebral failure	2.27 (1.55-3.31)	<0.001		
With ALSS	0.60 (0.39-0.95)	0.027	0.59 (0.38-0.94)	0.025

**Table 5:** Summary of the results of multivariate analyses of 28-day mortality in HBV-derived HRS patients after PSM who received ALSS versus SMT treatment with risk stratification by number of organ failures or AKI degree.

Analysis	Treatment	Crude model		Model 1		Model 2	
		HR (95% CI)	*P* value	HR (95% CI)	*P* value	HR (95% CI)	*P* value
AKI stage 1	ALSS (SMT as reference)	1.03 (0.60-1.79)	0.908	1.04 (0.60-1.80)	0.892	0.76 (0.43-1.35)	0.352
AKI stage 2	ALSS (SMT as reference)	0.41 (0.13-1.35)	0.143	0.47 (0.14-1.64)	0.238	0.24 (0.03-1.90)	0.175
AKI stage 3	ALSS (SMT as reference)	0.37 (0.16-0.88)	0.024	0.34 (0.14-0.83)	0.018	0.29 (0.12-0.70)	0.006
Organ failure (≤ 1)	ALSS (SMT as reference)	0.72 (0.36-1.43)	0.345	0.72 (0.36-1.43)	0.344	0.68 (0.32-1.43)	0.307
Organ failure (≥ 2)	ALSS (SMT as reference)	0.41 (0.23-0.74)	0.003	0.42 (0.23-0.76)	0.004	0.52 (0.28-0.95)	0.033

Model 1 was adjusted for age and sex. Model 2 was adjusted for age, sex, neutrophils, alanine aminotransferase (ALT), albumin, serum bilirubin, COSSH-ACLFs, and international normalized ratio (INR). AKI: acute kidney injury; SMT: standard medical treatment; ALSS: artificial liver support system.

**Table 6:** Patients characteristics before and post-ALSS treatment.

Variates	Pre-ALSS	Post-ALSS	*P* value
ALT (U/L)	147.00 (73.00-377.00)	103.00 (44.50-235.00)	0.120
Serum bilirubin (mg/dL)	20.07 (10.64-28.12)	7.51 (1.47-27.75)	0.004
Creatinine (mg/dL)	0.93 (0.71-1.61)	1.51 (0.70-2.36)	0.213
INR	2.08 (1.74-2.63)	2.21 (1.55-3.26)	0.533
Neutrophil (%)	73.50 (63.58-81.15)	83.60 (76.08-86.60)	0.068

ALT: alanine aminotransferase; INR: international normalized ratio.

**Table 7:** Univariate and multivariate analysis of the difference of variates between post- and pre-ALSS groups as risk factors on 28-day mortality in patients treated with ALSS.

Variates	Univariate Cox regression		Multivariate Cox regression	
	HR (95% CI)	*P* value	HR (95% CI)	*P* value
*Δ*Serum bilirubin	1.00 (1.00-1.00)	0.301		
*Δ*ALT	1.00 (1.00-1.00)	0.581		
*Δ*Neutrophil	0.99 (0.98-1.00)	0.075		
*Δ*INR	1.49 (1.12-1.98)	0.006	1.42 (1.06-1.90)	0.020
*Δ*Creatinine	1.00 (1.00-1.00)	0.003	1.00 (1.00-1.00)	0.016

ALT: alanine aminotransferase; INR: international normalized ratio.

## Data Availability

The original contributions presented in the study are included in the article/Supplementary Material; further inquiries can be directed to the corresponding authors.

## Supplementary Materials

Supplementary Materials Table S1: baseline characteristics of patients in different AKI stages. ALT: alanine aminotransferase; CLIF-SOFA: Chronic Liver Failure-Sequential Organ Failure Assessment; COSSH-ACLF: Chinese Group on the Study of Severe Hepatitis B-Acute-on-Chronic Liver Failure; HBV: hepatitis B virus; INR: international normalized ratio; MAP: mean arterial pressure; MELD: Model for End-Stage Liver Disease. Table S2: baseline characteristics of patients with organ failures. ALT: alanine aminotransferase; CLIF-SOFA: Chronic Liver Failure-Sequential Organ Failure Assessment; COSSH-ACLF: Chinese Group on the Study of Severe Hepatitis B-Acute-on-Chronic Liver Failure; HBV: hepatitis B virus; INR: international normalized ratio; MAP: mean arterial pressure; MELD: Model for End-Stage Liver Disease.
