# Supplementary material for: The Effect of Artificial Liver Support System on Prognosis of HBV-Derived Hepatorenal Syndrome: A Retrospective Cohort Study
Source: Dis Markers. 2022 Jun 1;2022:3451544. doi: 10.1155/2022/3451544 (PMC9177308; doi:10.1155/2022/3451544)
Supplement: Supplementary Materials — Table S1: baseline characteristics of patients in different AKI stages. ALT: alanine aminotransferase; CLIF-SOFA: Chronic Liver Failure-Sequential Organ Failure Assessment; COSSH-ACLF: Chinese Group on the Study of Severe Hepatitis B-Acute-on-Chronic Liver Failure; HBV: hepatitis B virus; INR: international normalized ratio; MAP: mean arterial pressure; MELD: Model for End-Stage Liver Disease. Table S2: baseline characteristics of patients with organ failures. ALT: alanine aminotransferase; CLIF-SOFA: Chronic Liver Failure-Sequential Organ Failure Assessment; COSSH-ACLF: Chinese Group on the Study of Severe Hepatitis B-Acute-on-Chronic Liver Failure; HBV: hepatitis B virus; INR: international normalized ratio; MAP: mean arterial pressure; MELD: Model for End-Stage Liver Disease. [file 3451544.f1.docx]

Table S1. Baseline characteristics of patients in different AKI stages.

| Variates | AKI stage 1 | | | AKI stage 2 | | | AKI stage 3 | | |
| --- | --- | --- | --- | --- | --- | --- | --- | --- | --- |
|  | SMT | ALSS | *P* value | SMT | ALSS | *P* value | SMT | ALSS | *P* value |
| n | 178 | 30 |  | 46 | 6 |  | 51 | 14 |  |
| Age (year) | 58.66 (12.06) | 57.27 (11.80) | 0.557 | 59.04 (10.61) | 48.33 (20.28) | 0.044 | 55.08 (11.87) | 48.36 (9.98) | 0.057 |
| Male sex | 133 (74.7) | 24 (80.0) | 0.695 | 32 (69.6) | 6 (100.0) | 0.275 | 40 (78.4) | 12 (85.7) | 0.821 |
| Degree of HE |  |  | 0.916 |  |  | 0.463 |  |  | 0.009 |
| Without HE (%) | 109 (61.2) | 20 (66.7) |  | 20 (43.5) | 2 ( 33.3) |  | 27 (52.9) | 1 ( 7.1) |  |
| I | 22 (12.4) | 4 (13.3) |  | 8 (17.4) | 0 ( 0.0) |  | 7 (13.7) | 2 (14.3) |  |
| II | 14 ( 7.9) | 1 ( 3.3) |  | 9 (19.6) | 1 ( 16.7) |  | 4 ( 7.8) | 5 (35.7) |  |
| III | 8 ( 4.5) | 1 ( 3.3) |  | 2 ( 4.3) | 1 ( 16.7) |  | 6 (11.8) | 4 (28.6) |  |
| IV | 25 (14.0) | 4 (13.3) |  | 7 (15.2) | 2 ( 33.3) |  | 7 (13.7) | 2 (14.3) |  |
| Ascitic (%) |  |  | 0.01 |  |  | 0.504 |  |  | 0.693 |
| Grade 1 | 29 (16.3) | 7 (23.3) |  | 11 (23.9) | 2 ( 33.4) |  | 10 (19.6) | 3 (21.4) |  |
| Grade 2 | 51 (28.7) | 14 (46.7) |  | 12 (26.1) | 3 ( 50.0) |  | 23 (45.1) | 6 (42.9) |  |
| Grade 3 | 65 (36.5) | 3 (10.0) |  | 13 (28.3) | 1 ( 16.7) |  | 8 (15.7) | 3 (21.4) |  |
| Missing data | 33 (18.5) | 6 (20.0) |  | 10 (21.7) | 0 ( 0.0) |  | 10 (19.6) | 2 (14.3) |  |
| MAP (mmHg) | 98.90 (17.44) | 94.48 (15.88) | 0.195 | 98.43 (15.81) | 104.67 (7.22) | 0.349 | 94.63 (14.80) | 98.69 (15.38) | 0.371 |
| HR | 86.65 (15.77) | 88.27 (13.00) | 0.595 | 88.87 (17.20) | 77.83 (9.58) | 0.132 | 85.22 (17.76) | 82.71 (14.71) | 0.631 |
| INR | 1.91 (0.76) | 2.24 (0.82) | 0.035 | 2.23 (1.06) | 1.93 (0.41) | 0.494 | 2.15 (0.77) | 2.39 (0.64) | 0.29 |
| Neutrophil (%) | 73.97 (12.61) | 73.52 (10.53) | 0.854 | 76.69 (10.79) | 63.03 (7.11) | 0.004 | 76.43 (11.53) | 71.45 (11.37) | 0.156 |
| Albumin (g/L) | 29.70 (5.24) | 29.37 (4.92) | 0.75 | 30.65 (5.33) | 29.77 (3.18) | 0.695 | 29.28 (5.89) | 29.82 (4.42) | 0.751 |
| Globulin (g/L) | 29.35 (8.50) | 30.71 (8.47) | 0.421 | 25.83 (7.99) | 27.37 (5.08) | 0.649 | 28.91 (11.55) | 27.11 (11.04) | 0.604 |
| ALT (U/L) | 123.98 (238.83) | 147.10 (160.65) | 0.61 | 108.02 (133.75) | 631.50 (503.10) | <0.001 | 274.29 (674.73) | 266.36 (351.87) | 0.966 |
| AST (U/L) | 169.92 (356.75) | 219.42 (388.06) | 0.534 | 140.36 (134.66) | 506.80 (445.17) | <0.001 | 308.18 (969.22) | 441.78 (477.50) | 0.691 |
| Hemoglobin (g/L) | 97.75 (27.24) | 109.10 (24.43) | 0.036 | 101.69 (22.32) | 124.67 (18.75) | 0.02 | 108.02 (27.37) | 123.86 (27.88) | 0.064 |
| Cystatin C (mg/L) | 2.56 (1.38) | 2.09 (1.10) | 0.201 | 2.27 (1.16) | 1.02 (0.25) | 0.027 | 2.27 (1.44) | 0.99 (0.23) | 0.018 |
| Urea (mmol/L) | 18.83 (12.88) | 14.76 (10.34) | 0.102 | 15.51 (10.93) | 4.42 (1.99) | 0.017 | 21.96 (40.79) | 4.84 (2.54) | 0.124 |
| Creatinine (mg/dL) | 212.81 (158.94) | 151.63 (118.90) | 0.045 | 144.07 (87.42) | 67.50 (13.98) | 0.039 | 185.55 (160.51) | 58.50 (13.44) | 0.005 |
| Serum bilirubin (mg/dL) | 233.50 (216.07) | 329.33 (197.27) | 0.024 | 386.09 (244.14) | 313.50 (98.08) | 0.478 | 304.45 (222.62) | 377.72 (235.93) | 0.285 |
| GGT (U/L) | 95.27 (109.61) | 76.30 (55.52) | 0.421 | 171.81 (430.67) | 99.80 (96.28) | 0.716 | 140.97 (141.01) | 86.88 (56.51) | 0.297 |
| Potassium (mmol/L) | 5.02 (9.68) | 4.29 (0.78) | 0.682 | 4.24 (0.94) | 4.28 (0.81) | 0.911 | 4.20 (0.85) | 3.95 (0.58) | 0.3 |
| Sodium (mmol/L) | 134.15 (7.44) | 134.10 (4.33) | 0.971 | 133.65 (6.01) | 137.67 (1.86) | 0.113 | 133.20 (5.89) | 136.00 (6.97) | 0.134 |
| MELDs | 26.89 (9.13) | 28.99 (7.15) | 0.233 | 28.71 (9.61) | 24.37 (2.51) | 0.28 | 28.92 (10.82) | 25.96 (6.43) | 0.335 |
| iMELD | 50.58 (11.25) | 52.30 (8.47) | 0.427 | 52.86 (12.06) | 42.50 (4.89) | 0.044 | 52.20 (11.91) | 45.27 (7.70) | 0.044 |
| CTP | 10.75 (1.97) | 11.47 (1.48) | 0.06 | 11.37 (1.90) | 12.17 (1.17) | 0.323 | 11.61 (1.76) | 12.43 (1.60) | 0.12 |
| CLIF-ACLFs | 48.92 (10.84) | 49.96 (8.67) | 0.618 | 52.32 (11.08) | 42.93 (10.43) | 0.055 | 50.31 (10.25) | 47.89 (11.04) | 0.444 |
| CLIF-SOFAs | 9.84 (3.66) | 10.47 (3.18) | 0.381 | 10.63 (3.56) | 10.50 (2.43) | 0.931 | 10.78 (4.20) | 10.43 (2.10) | 0.761 |
| COSSH-ACLFs | 6.99 (1.76) | 7.17 (1.59) | 0.606 | 7.75 (2.04) | 6.59 (1.15) | 0.18 | 7.31 (1.93) | 7.53 (1.48) | 0.693 |
| Liver failure | 81 (45.5) | 20 (66.7) | 0.051 | 33 (71.7) | 6 (100.0) | 0.316 | 31 (60.8) | 9 (64.3) | 1 |
| Coagulation failure | 33 (18.5) | 10 (33.3) | 0.108 | 12 (26.1) | 0 ( 0.0) | 0.362 | 14 (27.5) | 6 (42.9) | 0.436 |
| Cerebral failure | 33 (18.5) | 5 (16.7) | 1 | 9 (19.6) | 3 ( 50.0) | 0.251 | 13 (25.5) | 6 (42.9) | 0.35 |

ALT: Alanine aminotransferase; CLIF-SOFA: Chronic Liver Failure-Sequential Organ Failure Assessment; COSSH-ACLF: Chinese Group on the Study of Severe Hepatitis B-Acute-on-Chronic Liver Failure; HBV: Hepatitis B virus; INR: International normalized ratio; MAP: Mean arterial pressure; MELD: Model for End-Stage Liver Disease.

Table S2. Baseline characteristics of patients with organ failures.

| Variates | Organ failure (≤ 1) | | | Organ failure (≥ 2) | | |
| --- | --- | --- | --- | --- | --- | --- |
|  | SMT | ALSS | *P* value | SMT | ALSS | *P* value |
| n | 88 | 27 |  | 62 | 23 |  |
| Age (year) | 54.41 (11.09) | 53.85 (12.58) | 0.825 | 53.92 (12.35) | 53.52 (13.86) | 0.899 |
| Male sex | 72 ( 81.8) | 22 ( 81.5) | 1 | 52 (83.9) | 20 (87.0) | 0.99 |
| Degree of HE | |  | 0.512 |  |  | 0.136 |
| Without HE (%) | 64 ( 72.7) | 18 ( 66.7) |  | 17 (27.4) | 5 (21.7) |  |
| I | 12 ( 13.6) | 4 ( 14.8) |  | 12 (19.4) | 2 ( 8.7) |  |
| II | 5 ( 5.7) | 4 ( 14.8) |  | 2 ( 3.2) | 3 (13.0) |  |
| III | 4 ( 4.5) | 1 ( 3.7) |  | 5 ( 8.1) | 5 (21.7) |  |
| IV | 3 ( 3.4) | 0 ( 0.0) |  | 26 (41.9) | 8 (34.8) |  |
| Ascitic (%) | |  | 0.067 |  |  | 0.171 |
| Grade 1 | 16 ( 18.2) | 5 ( 18.5) |  | 14 (22.6) | 7 (30.4) |  |
| Grade 2 | 23 ( 26.1) | 14 ( 51.9) |  | 14 (22.6) | 9 (39.1) |  |
| Grade 3 | 33 ( 37.5) | 4 ( 14.8) |  | 19 (30.6) | 3 (13.0) |  |
| Missing data | 16 ( 18.2) | 4 ( 14.8) |  | 15 (24.2) | 4 (17.4) |  |
| MAP (mmHg) | 95.26 (15.89) | 94.91 (12.60) | 0.918 | 96.63 (16.37) | 99.19 (17.68) | 0.533 |
| HR | 84.06 (14.26) | 87.22 (14.04) | 0.313 | 92.76 (18.42) | 83.39 (12.72) | 0.027 |
| INR | 1.83 (0.53) | 1.86 (0.43) | 0.747 | 3.08 (1.20) | 2.69 (0.78) | 0.155 |
| Neutrophil (%) | 69.72 (13.48) | 70.63 (11.52) | 0.752 | 77.36 (11.41) | 72.92 (9.97) | 0.103 |
| Albumin (g/L) | 28.75 (5.51) | 29.38 (4.43) | 0.587 | 30.07 (5.87) | 29.74 (4.76) | 0.809 |
| Globulin (g/L) | 29.00 (7.91) | 29.59 (8.40) | 0.741 | 28.19 (9.64) | 28.96 (9.78) | 0.745 |
| ALT (U/L) | 88.61 (152.40) | 219.00 (346.58) | 0.007 | 275.23 (607.82) | 261.65 (275.19) | 0.918 |
| AST (U/L) | 127.76 (230.67) | 244.00 (293.48) | 0.062 | 402.45 (916.24) | 383.11 (531.78) | 0.933 |
| Hemoglobin (g/L) | 96.08 (24.40) | 112.00 (26.33) | 0.005 | 105.02 (28.10) | 118.87 (24.62) | 0.04 |
| Cystatin C (mg/L) | 2.36 (1.25) | 1.95 (1.17) | 0.288 | 2.81 (2.24) | 1.28 (0.65) | 0.014 |
| Urea (mmol/L) | 17.69 (14.92) | 11.50 (8.94) | 0.044 | 18.78 (13.94) | 9.84 (10.23) | 0.006 |
| Creatinine (mg/dL) | 198.50 (163.10) | 116.48 (57.60) | 0.012 | 204.22 (150.64) | 114.26 (139.06) | 0.015 |
| Serum bilirubin (mg/dL) | 195.75 (204.41) | 263.98 (195.67) | 0.128 | 399.62 (171.47) | 431.38 (162.96) | 0.444 |
| GGT (U/L) | 98.86 (130.23) | 88.28 (73.32) | 0.744 | 90.66 (91.42) | 75.56 (46.60) | 0.513 |
| Potassium (mmol/L) | 5.72 (13.83) | 4.13 (0.78) | 0.552 | 4.61 (1.10) | 4.27 (0.70) | 0.18 |
| Sodium (mmol/L) | 133.63 (8.87) | 134.37 (4.57) | 0.677 | 132.04 (5.55) | 135.87 (5.65) | 0.006 |
| MELDs | 25.27 (7.82) | 24.63 (6.06) | 0.7 | 36.49 (7.75) | 31.05 (5.81) | 0.003 |
| iMELD | 48.05 (10.81) | 46.73 (6.58) | 0.549 | 60.24 (9.95) | 52.00 (10.16) | 0.001 |
| CTP | 10.57 (1.83) | 11.00 (1.27) | 0.256 | 12.76 (1.39) | 12.78 (1.20) | 0.94 |
| CLIF-ACLFs | 43.55 (8.62) | 43.09 (7.23) | 0.802 | 58.90 (8.46) | 54.93 (8.16) | 0.056 |
| CLIF-SOFAs | 8.56 (2.80) | 8.63 (1.90) | 0.9 | 14.06 (2.59) | 12.61 (2.02) | 0.017 |
| COSSH-ACLFs | 6.32 (1.21) | 6.15 (0.77) | 0.503 | 9.33 (1.80) | 8.43 (1.22) | 0.03 |
| Liver failure | 29 ( 33.0) | 13 ( 48.1) | 0.228 | 55 (88.7) | 22 (95.7) | 0.578 |
| Coagulation failure | 6 ( 6.8) | 2 ( 7.4) | 1 | 44 (71.0) | 14 (60.9) | 0.531 |
| Cerebral failure | 7 ( 8.0) | 1 ( 3.7) | 0.744 | 31 (50.0) | 13 (56.5) | 0.772 |

ALT: Alanine aminotransferase; CLIF-SOFA: Chronic Liver Failure-Sequential Organ Failure Assessment; COSSH-ACLF: Chinese Group on the Study of Severe Hepatitis B-Acute-on-Chronic Liver Failure; HBV: Hepatitis B virus; INR: International normalized ratio; MAP: Mean arterial pressure; MELD: Model for End-Stage Liver Disease.
